# ^18^F-FLT PET/CT in Patients with Gastric Carcinoma

**DOI:** 10.1155/2013/696423

**Published:** 2013-12-25

**Authors:** Bogdan Małkowski, Tomasz Staniuk, Ewa Śrutek, Tomasz Gorycki, Wojciech Zegarski, Michał Studniarek

**Affiliations:** ^1^Department of Nuclear Medicine, Oncological Centre, Bydgoszcz, Poland; ^2^Department of Positron Emission Tomography and Molecular Diagnostics, Collegium Medicum, Nicolaus Copernicus University, 85-796 Bydgoszcz, Poland; ^3^Department of Oncological Surgery, Oncological Centre, 85-796 Bydgoszcz, Poland; ^4^Department of Cancer Pathology and Pathomorphology, Collegium Medicum, Nicolaus Copernicus University, 85-796 Bydgoszcz, Poland; ^5^Department of Radiology, Medical University of Gdańsk, 80-952 Gdańsk, Poland

## Abstract

The aim of the study was to evaluate the usefulness of 18F-FLT PET/CT in the detection and differentiation of gastric cancers (GC). 104 consecutive patients (57 cases of adenocarcinoma tubulare (G2 and G3), 17 cases of mucinous adenocarcinoma, 6 cases of undifferentiated carcinoma, 14 cases of adenocarcinoma partim mucocellulare, and 10 cases of end stage gastric cancer) with newly diagnosed advanced gastric cancer were examined with FLT PET/CT. For quantitative and comparative analyses, the maximal standardized uptake value (SUV_max_) was calculated for both the tumors and noninvaded gastric wall. *Results.* There were found, in the group of adenocarcinoma tubulare, SUV_max_ 1.5–23.1 (7.46 ± 4.57), in mucinous adenocarcinoma, SUV_max_ 2.3–10.3 (5.5 ± 2.4), in undifferentiated carcinoma, SUV_max_ 3.1–13.6 (7.28 ± 3.25), in adenocarcinoma partim mucocellulare, SUV_max_ 2–25.3 (7.7 ± 6.99), and, in normal gastric wall, SUV_max_ 1.01–2.55 (1.84 ± 0.35). For the level of 2.6 cut-off value between the normal wall and neoplasm FLT uptake from ROC analysis, all but five gastric cancers showed higher accumulation of FLT than noninfiltrated mucosa. *Conclusion.* Gastric cancer presents higher accumulation of 18F-FLT than normal, distended gastric mucosa. Significantly higher accumulation was shown in cancers better differentiated and with higher cellular density.

## 1. Introduction

Gastric cancer (GC) is an aggressive neoplasm with very poor prognosis. In Poland, in 2010, a number of 5364 people died (3486 men and 1878 women) due to gastric cancer. During the last 4 decades, both morbidity and mortality have dropped significantly in Poland from the 1st place at the beginning of the 1970s to the 4th most common cancer related death in men and the 7th in females [1]. The treatment of choice for GC is complete tumor resection. Early detection and surgery have improved the results of treatment. However, many patients are still diagnosed with advanced-stage disease. Accurate determination of local invasion, tumor size and location, lymph node involvement, and distant metastases is of great importance in the qualification of patients to adequate treatment.

Detection of early-stage GC by ^18^F-FDG PET is not successful, as FDG uptake is strongly related to tumor size, location, and histopathology, for example, a content of mucus [2, 3]. In 1998, ^18^F-FLT (FLT)—a new radiotracer with the potential ability to be captured by fast proliferating cells—was reported [4]. The authors found that FLT is accumulated in proliferating tissues by the action of thymidine kinase and is resistant to degradation. In PET, it produces high-contrast images of normal marrow and tumors in human. Gastric mucosa is also proliferating tissue, so it can be important whether mucosal FLT uptake can affect the detection of gastric cancer. Choice of unaffected gastric mucosa on the basis of PET only is doubtful, so in this work the PET/CT method was used to measure the FLT uptake within the normal gastric mucosa.

The purpose of the study was to verify the high potential to diagnose and differentiate GC using ^18^F-FLT and to elaborate real cut-off value for SUV_max⁡_ between the cancer tissue and normal gastric mucosa.

## 2. Material and Methods

104 consecutive patients (65 men and 39 women; mean age: 63 years) with the diagnosis of gastric tumor (biopsy-proven cancers) were included in this prospective study. They were enrolled to our department to diagnose the stage of the GC using FLT PET/CT. Written informed consent was obtained from all patients. The study protocol was approved by the local ethics committee of the Medical University Nicolaus Copernicus of Torun. In 10 patients with end-stage gastric cancer, the full verification of cancer type was not available.

The staging was not the issue of this paper. The aim of this evaluation was to compare uptake in normal and cancerous tissue in the stomach. The staging was described in our initial report published in June 2013 [5].

On the basis of histopathological evaluation of biopsy samples and/or removed specimens, the microscopic growth type in 94 patients was diagnosed and presented in Table 1. In some cases, there was no possibility to achieve full information about specific pathological classification. For example, there was group of patients in whom the surgery was canceled because of too advanced stage. In these patients, we have no full pathological information apart coming from endoscopy's specimens. Some frequently used classifications of diagnosed GC are presented in Table 1.

In order to assess the [^18^F]FLT uptake in the normal gastric wall the SUV_max⁡_ was measured in the area of normal gastric wall indicated on the basis of gastroscopy in 25 out of 104 patients in the study. The number of patients was limited to 25, as it was sufficient to further statistical analysis.

[^18^F]FLT was synthesized in our laboratory using R&D Syncrom module (Raytest) following the standard operating procedure (SOP). Radiosynthesis of [^18^F]FLT is based on [^18^F]fluoride displacement of a protected nosylate precursor. A simple three-step synthesis was used to prepare radiochemically pure [^18^F]FLT—98% ± 0.98%, at the end of synthesis within 45 min and with a 15% ± 7.6% radiochemical yield.

Patients fasted for at least 6 hours before the PET/CT scan. They were given antiperistalsis drug Buscopan (10 mg p.o.) 1 h before FLT injection.

Imaging was performed on the whole-body high-resolution PET/CT scanner Biograph 6. The images were acquired 60 min after administration of 350 ± 20 MBq of radiotracer. Standard CT scans were undertaken at 120 kV, 100 mAs, and 0.8 s rotation with a 1.25 mm slice width with no contrast injection. Pitch was 0.9. PET data were acquired in 3D mode for 3 min/bed. Acquisition of PET/CT was performed in two steps. Just before acquisition, patient drunk a glass (300 mL) of water to fulfill stomach. First, whole-body CT for attenuation correction and anatomical localization without contrast media was done. Immediately after CT, PET acquisition of two beds of placed on upper and mild abdomen was performed. As we tested before, this acquisition enables showing distended stomach without wall movement. The PET and CT parts of the image were exactly in the same position. Next, (without patient's position change) whole-body PET was performed. For attenuation correction and localization, the first whole-body CT was used.

Emission data were corrected for randomness, dead time, scatter, and attenuation.

## 3. Image Interpretation

To assure the proper interpretation, nuclear medicine and radiology specialists read the examination. They analyzed the image of the stomach using ^18^F FLT PET/CT and knowledge from gastroscopy. Any discrepancies in the interpretation were solved by consensus. They selected the tumor localization according to the CT. In the tumor, increased focal uptake was detected and assessed by measurements of the maximum standardized uptake value (SUV_max⁡_). The normal stomach wall was chosen in the area of thin stomach wall (CT) and uniform FLT uptake. Then, we assessed the physiological FLT uptake in the stomach wall having knowledge about localization of the cancer from gastroscopy descriptions (files). These areas were omitted in physiological FLT uptake analysis. The images from the first two beds covering only upper abdomen with distended stomach were taken into consideration in this analysis. The ellipsoidal (circular) VOIs with the diameter of 10 mm were placed in the chosen areas which were the most active (the highest uptake) in the areas affected by gastric cancer and free of disease. The SUV_max⁡_ from these VOI-s was calculated according to the standard formula (Bq/g × body weight (g)/injected activity in Bq).

The FLT uptake within the normal gastric wall was compared to accumulation within the tumor and the optimal cut-off value was estimated on the basis of ROC curve analysis.

The mean SUV_max⁡_ values were compared to cancer type (according to histopathological evaluation) and the differences were statistically tested in order to measure the capability of cancer differentiation.

## 4. Statistical Analysis

- SUV_max⁡_ values and the patients' age distribution were analyzed using Student's* t*-, Pearson, and Spearman tests. The age analysis was performed to assure lack of differences in the FLT uptake related to the age of patients.
- Differences in FLT accumulation were analyzed by the Mann-Whitney *U*-test.
- The changes of the sensitivities and specificities related to different thresholds between the SUV_max⁡_ values in normal gastric wall and cancer tissue were performed on the basis of ROC curve analysis.
- Differences were considered statistically significant at the *P* < 0.05 level.

## 5. Results

### 5.1. FLT Uptake in Normal Gastric Wall

**Figure 1:**
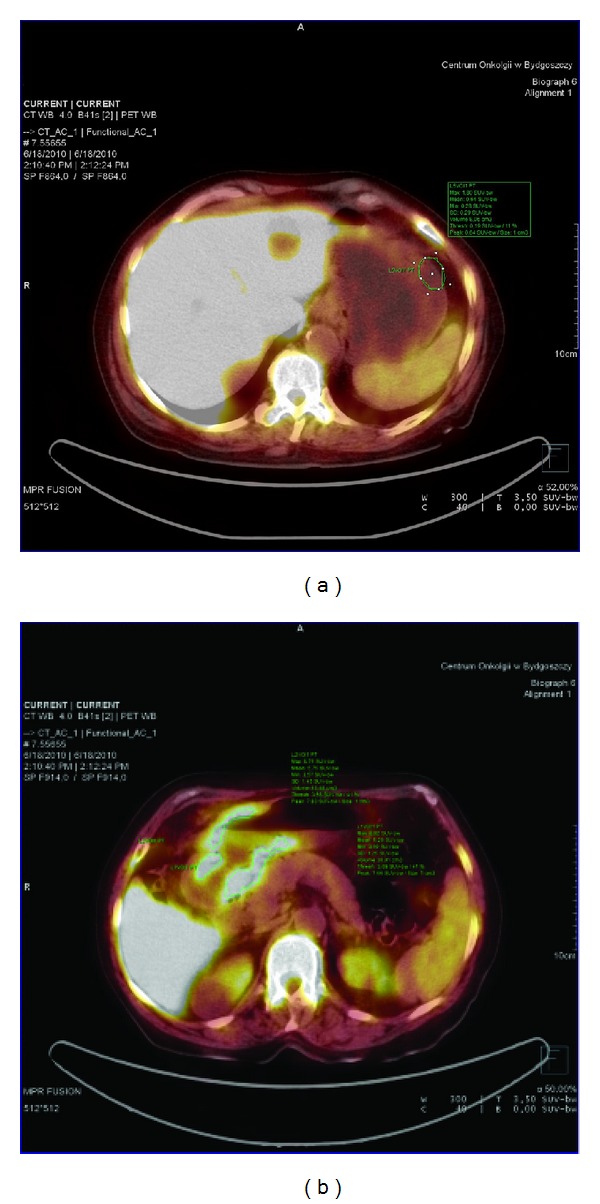
(a) ^18^FLT-PET in patient with undifferentiated adenocarcinoma. SUV_max⁡_ in normal gastric wall was 1.8. (b) ^18^FLT-PET in patient with undifferentiated adenocarcinoma. SUV_max⁡_ = 8.9.

In 25 out of 104 patients in the study, the SUV_max⁡_ was measured in normal gastric wall. On the basis of endoscopic evaluation and surgical estimation, the gastric wall with no infiltration was chosen (CT), and the SUV_max⁡_ was calculated. It has reached mean value and standard deviation of 1.84 ± 0.35, respectively (from 1.01 to 2.55) (Figure 1). There were no statistical differences in the FLT uptake in normal gastric mucosa related to age. The FLT uptake was significantly lower than that in the cancer tissue (mean SUV_max⁡_—7.27 ± 4.73) and the optimal cut-off value differentiating tumor versus nontumor SUV_max⁡_ was found at the level of 2.3 (sensitivity—97% and specificity—92%). For the threshold 2.6, the respective values were 94.7% and 100%, but in 5 patients with cancer the SUV_max⁡_ was below this value (false negative ratio—5.3%).

All the 5% of false negative cases but one were mucus containing cancers. To achieve the specificity, we have compared cancerous localization as described in the endoscopy and confirmed in the pathology report and normal gastric wall. There were no additional foci of increased uptake apart from true GC localizations. It means that we had no FP results.

### 5.2. FLT Uptake in Different Types of Gastric Cancer

**Figure 2:**
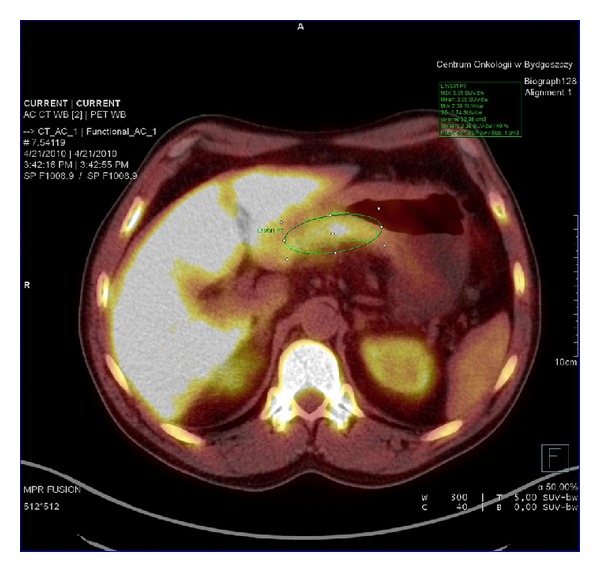
^18^FLT-PET in patient with mucinous adenocarcinoma. SUV_max⁡_ = 5.9.

**Figure 3:**
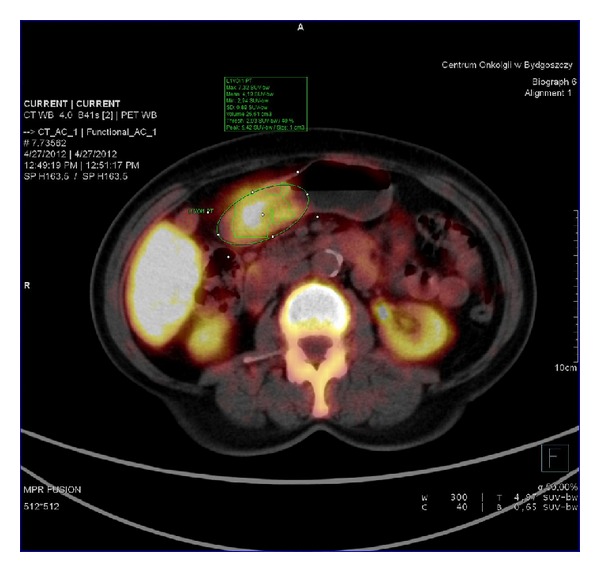
^18^FLT-PET in patient with adenocarcinoma tubulare G2 (G3). SUV_max⁡_ = 7.32.

SUV_max⁡_ of mucinous carcinoma presented in Table 2 was significantly different from other types of GC (Figures 2 and 3).

When the patients were divided according to Lauren classification into two groups and according to grading on G2 and G3 groups, other statistically significant differences of FLT uptake were found only for the G-grading. The results are presented in Table 3.

## 6. Discussion

### 6.1. Cancer Detection

Detection of gastric cancer is typically by endoscopy, and imaging is used to stage the disease rather than screening. In small tumors, usually endoscopic ultrasound is applied to stage cancer, but in larger ones it can be limited. Percutaneous ultrasound after careful preparation can be very valuable method in detection and characterization of digestive tract tumors, but assessment of whole stomach wall is extremely difficult [6, 7]. Computed tomography and MR imaging are used in locoregional nodal staging, but the full postsurgical verification is needed, not only the presence of lymph nodes on CT/MR images. After positive pathomorphological evaluation of tissue samples taken during endoscopy, there is a need to assess the real limits of tumor. ^18^F-FDG was found to have extremely different accumulation in different gastric cancer. It is believed that ^18^F-FLT can help in the problem, but further careful evaluation of its uptake in gastric cancer and additionally gastric nonneoplastic pathologies is still needed.

94 analyzed cases form the large group of patients with gastric cancer examined with ^18^F-FLT PET when compared to other publications. The quantitative measurements of SUV_max⁡_ are less dependent on ROI or VOI choice than the measurements of mean SUV value. Gastric mucosa is the tissue with very high proliferation. In normal conditions, its thickness can reach more than 10 mm, so, to avoid the false positive results, it should be significantly distended. The method of gastric wall distention is common and is realized by water or water-based contrast agents [8] or gas after ingestion of effervescent granules with a small amount of water [9]. Additionally, the digestive track peristalsis is suppressed by antiperistaltic drugs [8, 9]. In our group, there was the Buscopan tablet (10 mg) taken 1 hour before FLT injection. The SUV_max⁡_ of FLT within the distended normal gastric mucosa was found within the range of 1.01–2.55 (mean: 1.84). It was high enough to create 5% of FN results, when we put the cut-off value at the level of 2.6 (to avoid FP results). One can suppose that it is acceptable price for the possibility to use FLT PET in TNM staging; however, in five gastric cancers, SUV_max⁡_ was lower than 2.6. All of these cases but one were diagnosed as adenocarcinoma mucocellulare or partim mucocellulare.

### 6.2. Cancer Differentiation

The ^18^F-FDG PET is the most frequently used radiopharmaceutical for diagnosing the cancer. But it is well known that FDG uptake depends on some tissue properties, not specific for the malignant neoplasm only [10]. Kawamura et al. [11] analyzed GLUT1 protein expression in 617 carcinomas and 50 tubular adenomas of the stomach. None of the adenomas expressed GLUT1, whereas 182 of the 617 carcinomas (30%) were positive for GLUT1 expression. Furthermore, signet-ring cell carcinoma and mucinous adenocarcinoma showed very low positive values for GLUT1 expression (2 and 6%, resp.). Among the other histological types, papillary adenocarcinoma (44%) showed slightly higher positive values for GLUT1 expression than tubular (32%) or poorly differentiated adenocarcinoma (28%). Yamada et al. [12] evaluated the association between FDG uptake and histopathological type in 40 patients with gastric carcinoma among whom only 19 patients (48%) showed detectable FDG uptake. Cohesive carcinomas (papillary adenocarcinoma, tubular adenocarcinoma, and solid-type poorly differentiated adenocarcinoma) were significantly better detectable than noncohesive carcinomas (signet-ring cell carcinoma and nonsolid type poorly differentiated carcinoma −65% versus 14%, resp.). In 2007, Herrmann et al. [13] published paper in which they found 100% sensitivity in the diagnosis of gastric cancer with [^18^F]FLT (3′-Fluoro-3′deoxythymidine-) PET. They have compared the accumulation of FLT and FDG in gastric cancer and showed that FLT PET was more sensitive than FDG PET, especially in tumors with no or low FDG uptake. This publication opened the new era in the stomach cancer PET diagnostics, but the authors concentrated only on the primary advanced tumors. Presented above data and results of other studies suggest that FDG is not ideal tracer for this type of cancers.

Analyzing the pathomorphological cancer structure, we compared independently some present or absent features within the cancer types. Stahl et al. [3] in publication on usefulness of ^18^F-FDG PET in the diagnosis of gastric cancer concluded that FDG uptake is lower in nonintestinal than in intestinal Lauren type tumors. They described that only 60% of locally advanced GC were detected by FDG PET, but even 83% of intestinal type tumors were PET positive, when only 41% of diffused ones were seen. We found also lower accumulation of ^18^F-FLT in nonintestinal gastric cancers—mean SUV_max⁡_ = 6.84 versus 7.69 found in intestinal type tumors, but the difference was not statistically significant. It can suggest that cell proliferation is higher in intestinal GC Lauren type. The results Stahl et al. described for FDG PET were the same as those we found for FLT PET, showing higher uptake in nonmucus-containing GC with FLT SUV_max⁡_ = 7.27 versus 5.50 in mucinous adenocarcinoma. In this work, FDG SUV_max⁡_ was 7.2 in nonmucinous neoplasms versus 3.9 in mucus-containing ones. It can show that not only metabolic increase characterizes solid GC but higher cell proliferation as well. The uptake of both FDG and FLT was also higher in low-grade GC than in high-grade cancers. The differences were statistically significant for both radiopharmaceuticals (SUV 7.4 versus 5.2 for FDG and SUV_max⁡_ 8.2 versus 6.57 in G2 and G3 tumors, resp.). We showed that detection of GC using ^18^F-FLT PET was successful in 89 out of 94 patients (95% sensitivity) with 5 false negative cases (i.e., below SUV_max⁡_ threshold of 2.6). In these 5 cases, the FLT uptake was within the range of noninvaded gastric wall, while, in all others, there was a great variety of SUV_max⁡_ values. Only tumors with relatively lower cellular density (mucinous type) accumulated FLT significantly less than other ones. Histopathological types of gastric cancer donot reflect their ability to FLT accumulation which was supposed to be strongly related to cellular proliferation within the tumors. Kameyama et al. [14] in his study performed prospectively in 21 patients with advanced gastric cancer showed that the sensitivity in the diagnosis of gastric cancer with FLT and FDG was similar, but the mean SUV_max⁡_ for FLT (7 ± 3.3) was significantly lower than that for FDG (9.4 ± 6.3) (similar to results of Kim et al. [9]). The accumulation of FLT was significantly higher in high-grade gastric tumors (SUV_max⁡_ 8.5 versus 5.3 in low-grade ones) even if it did not correlate with KI-67 index. This was not the case in FDG uptake, but the number of patients enrolled into the study counted only 21 patients. High-grade tumors are frequently necrotic, so the mean cellularity can be lower in low-grade cancers. In our paper, we showed that FLT SUV_max⁡_ was significantly lower in cancers with lower cellular density—similar to results of Kameyama group. FLT uptake has been shown to be lower in cancer with higher mucinous content and probably lower cellularity (18.1% of patients with mucinous adenocarcinoma) but simultaneously higher in better differentiated tumors. It is suspected that FLT uptake is not a simple result of proliferation alone, but can use an additional mechanism related to nucleoside transporters. It should be carefully studied in further works. Due to possibility to indicate a low cohesive cancer type, we hope it can be useful in predicting prognosis, planning treatment, and monitoring response in patients with gastric cancer.

## 7. Conclusions

We found that all gastric cancer types presented higher mean accumulation of FLT when compared to noninfiltrated gastric wall as measured by SUV_max⁡_. The optimal cut-off value between the cancer and mucosal accumulation was found at the level of 2.6 with sensitivity of 95% and an acceptable FN ratio of 5%. FLT PET/CT accumulation is significantly higher in regions of higher cellular density and cellular proliferation as well as in better differentiated neoplasm.

## Figures and Tables

**Table 1:** Histopathology of diagnosed gastric cancers.

Histopathology	*N*	Total
AdenoCa tubulare	57	94
Mucinous adenoCa	17	
Undifferentiated carcinoma	6	
AdenoCa partim mucocellulare	14	

Lauren type intestinal	38	88
Lauren type nonintestinal	50	

G2	28	78
G3	50	

**Table 2:** The mean value of SUV_max_ (SD) in normal mucosa and different cancer types.

Type	*N*	Min SUV_max_	Max SUV_max_	Mean (SD)
Normal mucosa	25*	1.01	2.55	1.84 (0.35)**
AdenoCa tubulare	57	1.5	23.1	7.46 (4.57)
Mucinous adenoCa	17	2.3	10.3	5.50 (2.40)**
Undifferentiated carcinoma	6	3.1	13.6	7.28 (3.25)
Adenocarcinoma partim mucocellulare	14	2	25.3	7.7 (6.99)

All	94	2.0	25.3	7.27 ± 4.73

*The patients were the same.

**Significantly differs from other cancer types (*P* < 0.05).

**Table 3:** The mean value of SUV_max_ (SD) in different cancer types, as divided according to Lauren classification and grading, compared to whole population studied and between the individual groups.

No.	Type	*N*	Mean (SD) SUV_max_	Between groups
1	Lauren intestinal	38	7.69 (4.95)	*P* = 0.16
2	Lauren non-intestinal	50	6.84 (4.64)	1 versus 2
3	G2	28	8.2 (5.41)	*P* = 0.026*
4	G3	50	6.57 (4.59)	3 versus 4

*In G3 cancers the mean SUV_max_ value was significantly lower than in G2 tumors.
